# Fibrous Hamartoma of Infancy of the Arm Mimicking a Vascular Malformation: A Diagnostic Pitfall

**DOI:** 10.1055/a-2790-2093

**Published:** 2026-01-31

**Authors:** Layth J. M. Saada, Malak Ismael Marei, Izzeddin A. Bakri, Jamil Saada

**Affiliations:** 1 Faculty of Medicine and Health Sciences, Al-Quds University, Abu Dis, Jerusalem, State of Palestine; 2 General Surgery Department, Al-Mezan Speciality Hospital, Hebron, West Bank, State of Palestine; 3 Pathology Department, Makassed lslamic Chritable Hospital, Makassed Hospital, Jerusalem, Jerusalem District, State of Palestine; 4 Pediatric Surgery Department, Al-Mezan Speciality Hospital, Hebron, West Bank, State of Palestine; 5 Department of Pediatric Surgery, Al-Quds University, Abu Dis, Jerusalem, State of Palestine

**Keywords:** fibrous hamartoma of infancy, pediatric soft tissue tumor, triphasic histology, subcutaneous arm mass, pediatric surgery

## Abstract

**Background:**

Fibrous hamartoma of infancy (FHI) is a rare benign soft tissue tumor of early childhood, often misdiagnosed due to its clinical and/or radiological resemblance to vascular malformations or pediatric soft tissue neoplasms.

**Case Presentation:**

A 7-month-old male presented with a rapidly enlarging, firm, non-pulsatile subcutaneous mass involving the anterior aspect of almost the entire right arm. MRI suggested a low-flow vascular malformation; however, due to clinical concern for alternative pathology and the lesion's benign appearance, large size, superficial location, and resectability, complete excision was performed. Histopathology revealed the characteristic triphasic pattern confirming fibrous hamartoma of infancy. The patient recovered well with no recurrence at 3-month follow-up.

**Conclusion:**

This case highlights the diagnostic pitfalls of FHI, which may closely mimic vascular anomalies on imaging, and underscores the importance of surgical excision for both definitive diagnosis and curative treatment. To our knowledge, this represents the first reported case of FHI from Palestine.

## Introduction

Fibrous hamartoma of infancy (FHI) is a rare benign soft tissue tumor that typically occurs within the first 2 years of life, with nearly 91% of cases identified during the first year. 1 Clinically, it presents as a solitary, firm, and rapidly enlarging subcutaneous mass, most frequently affecting the axillae, back, or upper arms. 2 Its features may mimic a broad range of pediatric soft tissue tumors and vascular malformations, including vascular granulation, lymphatic malformation, fibromatosis, juvenile fibromas, and even sarcomas. 3 Radiologic findings are often suggestive yet nonspecific, underscoring the importance of histopathology. Characteristically, FHI exhibits a distinctive triphasic histological pattern composed of fibroblastic tissue, primitive mesenchyme, and mature adipose tissue, which serves as a key diagnostic hallmark. 2

Here, we present the case of a 7-month-old infant with a right arm mass, initially suspected to represent a vascular or lymphatic malformation but ultimately confirmed as FHI following surgical excision. This report highlights the diagnostic pitfalls associated with this entity and emphasizes the role of surgical excision as both a diagnostic and therapeutic approach.

## Case Presentation

**Fig. 1:**
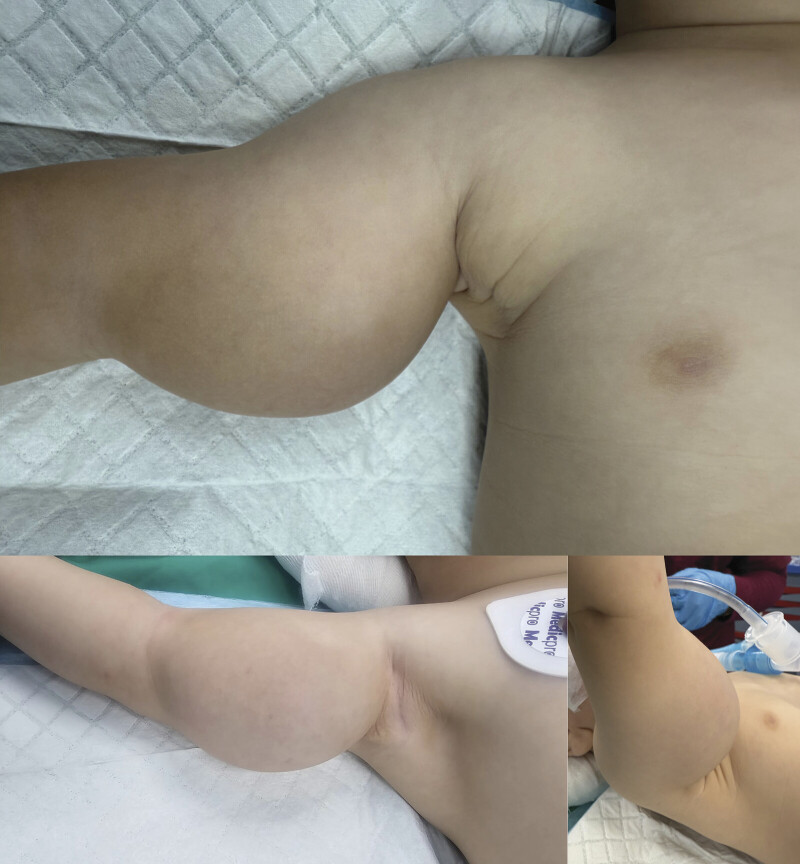
Large subcutaneous swelling of the right arm (approximately 7–8 cm).

A 7-month-old male infant presented with a progressively enlarging swelling over the anterior aspect of the entire right arm ( Fig. 1 ), first noted at the age of 4 months. There was no history of skin rash, fever, bleeding, discharge, or functional impairment of the affected limb. The infant was delivered at term via spontaneous vaginal delivery without antenatal or perinatal complications. Family history was negative for vascular malformations, congenital anomalies, or similar conditions.

On physical examination, the infant was active and developmentally appropriate for age. Vital signs were within normal limits. A firm, non-tender, non-pulsatile subcutaneous swelling was noted over the anterior aspect of almost the entire right arm, measuring approximately 7 to 8 cm at its greatest dimension ( Fig. 1 ). The overlying skin was intact with no erythema, discoloration, or ulceration. There was no regional lymphadenopathy. Distal neurovascular examination was normal. Apart from a mild pectus excavatum deformity of the anterior chest wall, no other systemic abnormalities were noted.

**Fig. 2:**
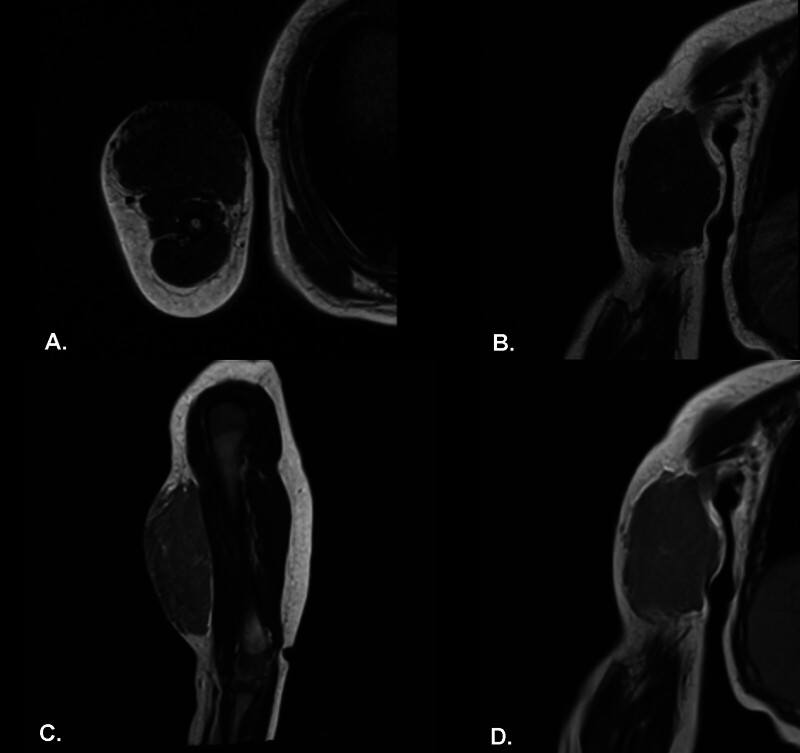
Axial T1-weighted ( **A** ), coronal T1-weighted ( **B** ), sagittal T2-weighted ( **C** ), and coronal T2-weighted ( **D** ) MR images of the right arm (non–fat suppressed) demonstrating a well-defined subcutaneous fusiform lesion with heterogeneous signal intensity. No frank intralesional fat streaks, intramuscular extension, or cortical invasion are identified.

Magnetic resonance imaging (MRI) of the right arm with intravenous contrast ( Figs. 2 3 4 5 ) revealed a well-defined fusiform subcutaneous heterogeneous mass occupying the anterior surface of almost the entire right arm, measuring approximately 6 × 4 cm. The lesion appeared hypointense on T1-weighted images and isointense on T2-weighted images, with progressively vivid enhancement beginning at 40 seconds. Few small internal cystic changes were present. Mild marrow edema was observed in the adjacent mid-humerus, although no frank cortical invasion or intramuscular extension was noted. The initial radiological impression favored a low-flow vascular malformation, most likely of venous origin, while a hemangioma or a microcystic-type lymphatic malformation was considered less likely.

**Fig. 3:**
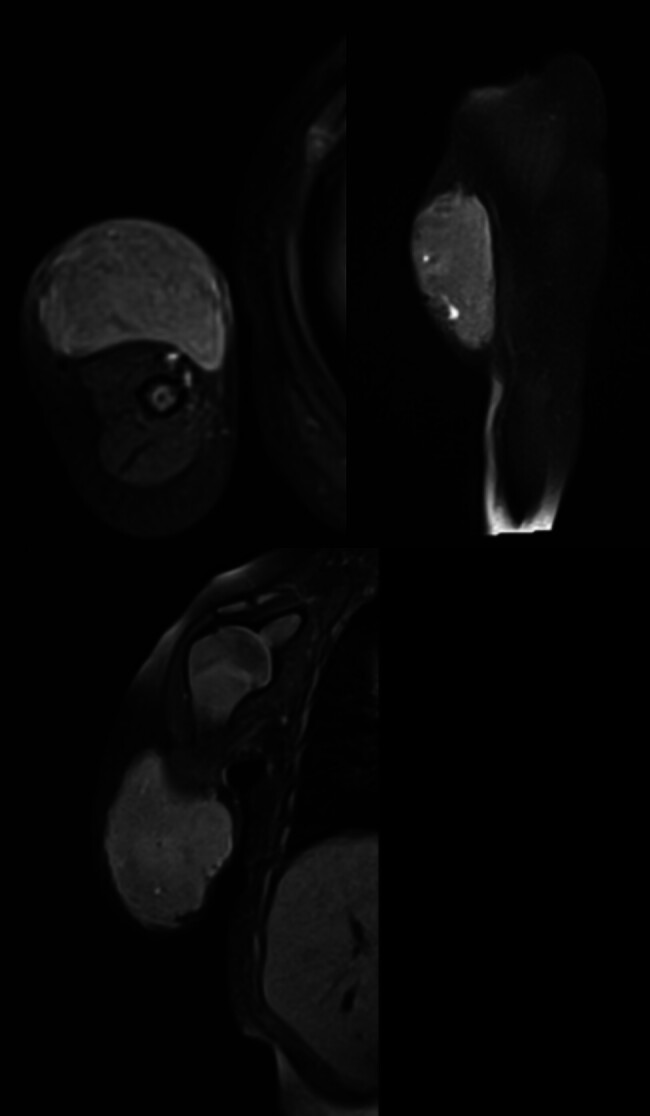
Axial, sagittal, and coronal fat-suppressed proton density MR images of the right arm showing a well-defined subcutaneous fusiform lesion with heterogeneous signal intensity. No frank intramuscular extension or cortical invasion is evident.

**Fig. 4:**
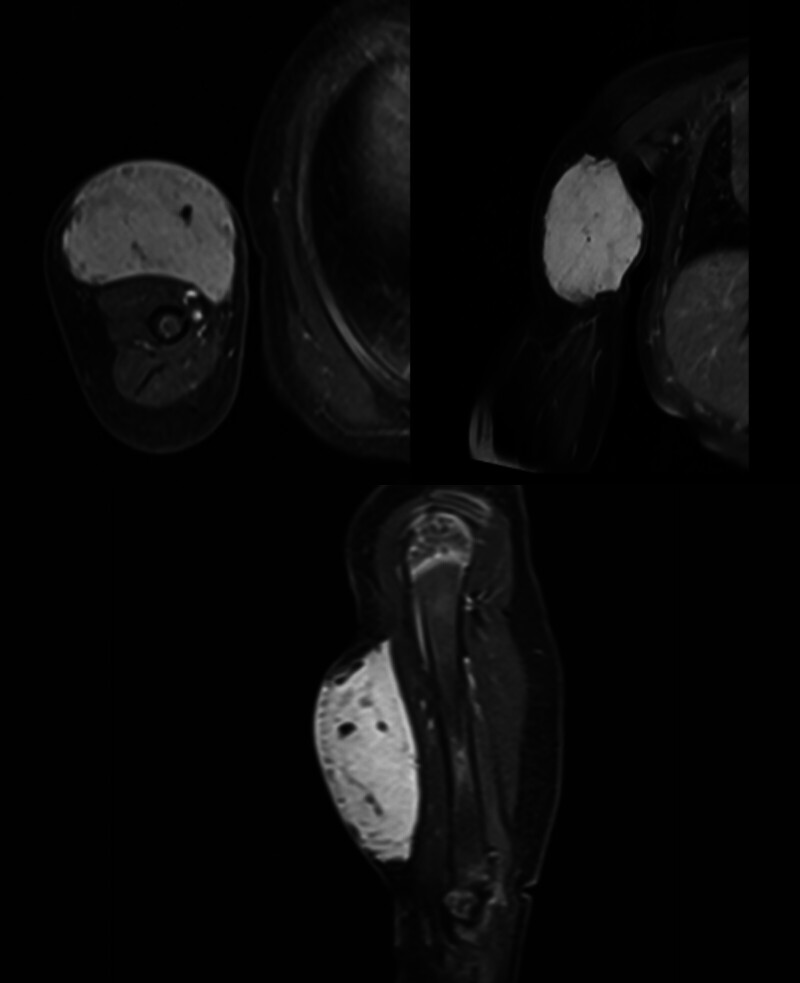
Axial, sagittal, and coronal post-contrast fat-suppressed T1-weighted MR images of the right arm demonstrating a well-defined subcutaneous fusiform lesion with vivid heterogeneous enhancement. No frank intramuscular extension or cortical invasion is identified.

**Fig. 5:**
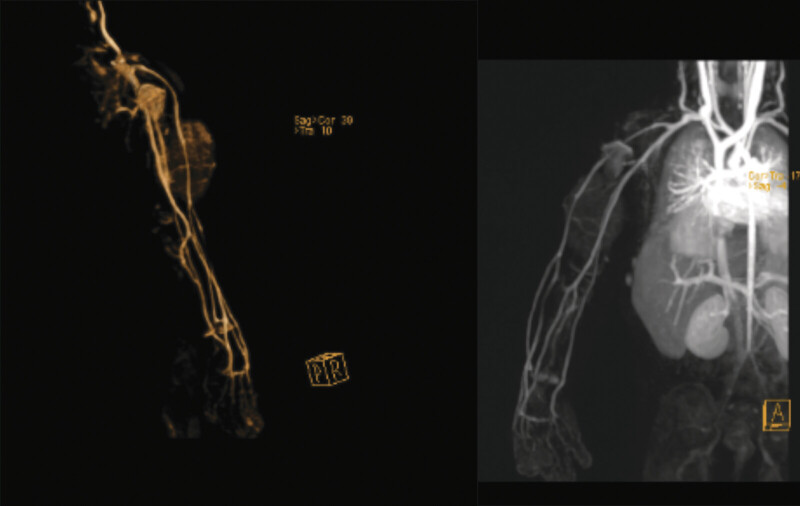
MR angiography of the right upper limb demonstrating preserved vascular anatomy without evidence of encasement or invasion by the subcutaneous lesion.

In view of the reported radiological findings, potential therapeutic options were discussed with the parents. However, based on the surgical team's clinical experience, review of the imaging, and careful clinical examination, there was significant concern for an alternative pathology. Surgical excision was therefore recommended as the most definitive and reliable management strategy. The parents agreed, and the patient was admitted for elective excision. Given the lesion's well-circumscribed, superficial, and technically resectable nature, despite its large size, complete excision was deemed the most appropriate approach.

**Fig. 6:**
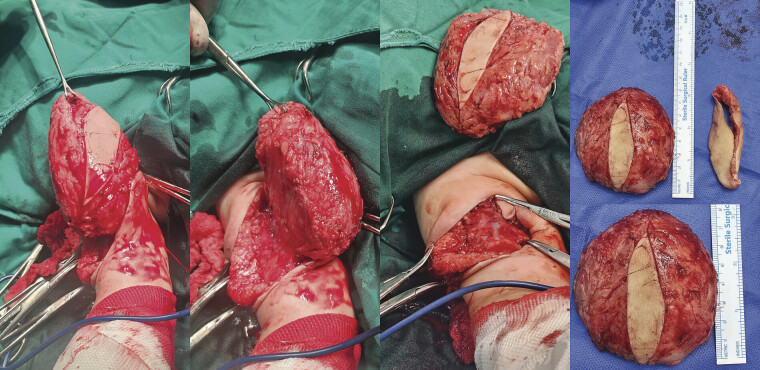
Intraoperative views and gross specimen showing en bloc excision of the large subcutaneous mass from the right arm (measuring approximately 7.5 × 8.0 cm), along with a cuff of redundant overlying skin.

Baseline laboratory investigations, including complete blood count and coagulation profile, were within normal limits. Surgical excision was performed under general anesthesia. A vertical elliptical skin incision was made over the lesion. Intraoperatively, the mass was subcutaneous, moderately adherent to the overlying skin, and supplied by two small feeding arteries, which were identified and ligated. Dissection was extended to the deep aspect of the lesion overlying the muscle, and the mass was excised en bloc along with a cuff of redundant overlying skin ( Fig. 6 ). The excised specimen measured approximately 7.5 × 8.0 cm. Hemostasis was secured, and the wound was closed in layers without drain placement. The specimen was sent for histopathological examination. The patient had an uneventful recovery and was discharged the following day on oral analgesics and antibiotics.

**Fig. 7:**
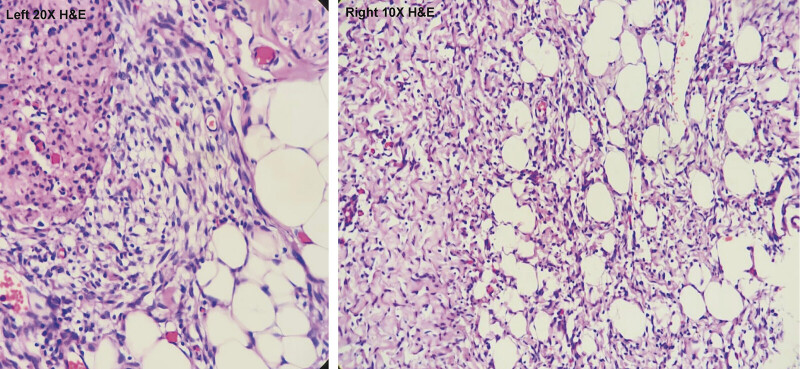
Low-power view (10 × , right) and higher-power view (20 × , left) showing a characteristic triphasic pattern composed of interlacing fibrous trabeculae, immature mesenchymal tissue with small spindle and stellate cells in a myxoid background, and interspersed mature adipose tissue. The fibrous component merges imperceptibly with the surrounding subcutaneous fat. No cytologic atypia, necrosis, or abnormal mitotic activity is seen.

Histopathological examination ( Fig. 7 ) revealed a poorly demarcated subcutaneous spindle-cell proliferation composed of intersecting fascicles of bland fibroblasts and myofibroblasts within a collagenous stroma. Variably sized nests of primitive, immature-appearing mesenchyme in a myxoid background, along with entrapped lobules of mature adipose tissue, were identified. No overt atypia, brisk mitotic activity, or necrosis was observed. Immunohistochemistry demonstrated patchy CD34 positivity, with negative staining for B-catenin, STAT-6, and S100. These features were consistent with fibroblastic/myofibroblastic proliferation with a triphasic pattern, favoring a fibrous hamartoma of infancy. The lesion measured 7 cm and was almost completely excised, with the closest margin 2 to 4 mm from the resection edge.

**Fig. 8:**
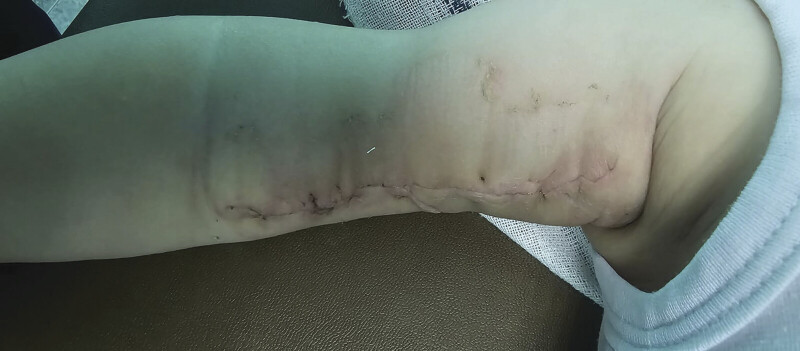
Postoperative photograph at 2-week follow-up showing well-healed incision without complications.

At 2-week follow-up, the patient demonstrated good wound healing ( Fig. 8 ). He remained well at 3 months with no evidence of recurrence.

## Discussion

FHI is a rare benign fibro-myofibroblastic lesion of the lower dermis and subcutis, with most cases presenting in the first year of life. 1 The entity was initially alluded to by Stout in 1954 as part of the spectrum of juvenile fibromatoses but was first clearly delineated by Reye in 1956, who described six infants under the term “subdermal fibromatous tumors of infancy.” Less than a decade later, Enzinger (1965) introduced the designation fibrous hamartoma of infancy and characterized its pathognomonic triphasic histology in a series of 30 cases. Since then, fewer than 200 to 250 cases have been reported worldwide, and, to our knowledge, this is the first documented case in Palestine. 4 5 6

FHI demonstrates a male predominance with a reported ratio of 2.4:1. Clinically, it usually presents as a painless, firm, and ill-defined subcutaneous mass, sometimes accompanied by overlying skin changes such as hyperpigmentation or increased coarse hair. 7 8 The most common anatomical sites are the axilla (17%), back (16%), and upper arm (14%), followed by the scrotum (9%). Less frequent locations include the chest wall, thigh, neck, breast, forearm, abdominal wall, buttocks, cheek, foot, shoulder, finger, scalp, flank, hip, and orbit. 7 8 Reported tumor sizes range from 1 to 8 cm in most cases but may reach up to 20 cm in diameter. Although the lesion may initially grow rapidly, spontaneous growth arrest is typically observed by the age of 5 years if left untreated. 9

Our patient's lesion was enlarging, subcutaneous, firm, and nonpulsatile, with intact overlying skin. MRI revealed a well-defined, heterogeneous, avidly enhancing lesion without frank intramuscular invasion. Based on these findings, the initial radiologic impression favored a low-flow vascular malformation, with a lymphatic malformation considered less likely. However, several clinical features made a vascular lesion less convincing and prompted surgical excision rather than alternative treatments such as sclerotherapy. These included: (i) firm rather than compressible consistency, (ii) absence of skin discoloration or phlebectasia, (iii) lack of bruit or thrill, (iv) tethering of the lesion to the overlying skin, and (v) rapid enlargement, which is more typical of high-flow vascular lesions and discordant with the low-flow pattern suggested radiologically. Such diagnostic misdirection toward vascular anomalies has been described in the literature, particularly when FHI occurs at atypical sites or when MRI lacks the classic fat signal, leading to confusion with vascular malformation, hemangiomas, neurofibromatosis, or fibromatosis. 10 Rare malignant entities such as infantile fibrosarcoma may be considered in the radiologic differential of enlarging soft-tissue masses in infancy, although no imaging features in our case suggested malignancy.

On MRI, FHI typically demonstrates fatty elements that appear hyperintense on T1-weighted and intermediate-to-high signal on T2-weighted images, while the fibrous elements remain hypointense on both sequences. Hemangiomas, in contrast, usually demonstrate striking T2 hyperintensity, rapid enhancement, infiltrative margins, and often with interspersed fat. The observed T2 hyperintensity in hemangiomas helps distinguish them from FHI. 9 11 In our case, several features supported the possibility of FHI, including the fusiform subcutaneous morphology, isointense rather than markedly hyperintense T2 signal, and delayed progressive vivid enhancement after 40 seconds. However, no frank intralesional fat streaks were observed on non–fat-suppressed T1-weighted images, which reduced radiologic specificity and precluded a confident preoperative diagnosis. In addition, the presence of small internal cystic changes and mild marrow edema in the adjacent humerus raised suspicion for a low-flow vascular malformation, more likely venous than lymphatic in origin, although the overall MRI findings were not fully consistent with a classic venous malformation. These overlapping yet inconclusive findings reinforced the decision for surgical excision, both to secure a definitive histopathological diagnosis and to provide curative treatment.

Histology remains the gold standard for diagnosis. FHI demonstrates a characteristic triphasic pattern composed of: (i) hypercellular fascicles of fibroblasts and myofibroblasts infiltrating adjacent fat, (ii) primitive myxoid mesenchymal tissue with delicate capillary networks, and (iii) mature adipose tissue. 8 Immunohistochemically, FHI shows patchy CD34 positivity within the primitive mesenchymal/spindle cell component, while negative β-catenin staining helps distinguish it from desmoid-type fibromatosis. Negative STAT6 excludes solitary fibrous tumor, and negative S100 highlights the adipocytic component. 7 12 13 In our case, the presence of the triphasic pattern together with patchy CD34 positivity and negative β-catenin, STAT6, and S100 confirmed the diagnosis.

Management of FHI typically consists of surgical excision, which provides both definitive diagnosis and curative treatment. In our case, excision was chosen after a broad differential diagnosis. Although the initial radiologic impression favored a vascular or lymphatic malformation, surgical excision remained an appropriate option for this well-circumscribed lesion, and the clinical reassessment, which suggested a higher likelihood of an alternative soft-tissue process, further supported proceeding with definitive removal. Excisional biopsy was preferred over incisional or needle biopsy because the lesion was well-defined, benign-appearing, and lacked invasive features, yet was large, disfiguring, and occupied most of the infant's arm. Given its superficial location and resectability, complete excision was deemed the most appropriate management. Recurrence is uncommon, reported in approximately 15% of cases, and usually results from incomplete excision or positive/close margins. Sclerotherapy, chemotherapy, and radiotherapy have no established role in treatment. 14 In our case, the lesion was completely excised, and the patient remained well with good healing and no recurrence up to 3-month follow-up.

This case underscores the value of correlating imaging with bedside findings to narrow the differential and emphasizes that definitive management should be based on integrated clinical–radiologic assessment and surgical judgment rather than imaging alone. In clinical practice, common conditions often dominate diagnostic reasoning, which may inadvertently lead to overlooking rarer entities—even when subtle but meaningful clues point toward them.

## Notes

Importance for the Pediatric Surgeon Fibrous hamartoma of infancy can closely mimic vascular or lymphatic malformations, leading to potential misdiagnosis. Awareness of this entity is essential to avoid inappropriate management, as complete surgical excision is both diagnostic and curative with excellent outcomes.
